# Male Dromedary Reproductive Emergencies: Clinical Presentation, Diagnosis, Management and Prognosis

**DOI:** 10.3390/ani16121843

**Published:** 2026-06-15

**Authors:** Ahmed Ali, Derar Derar

**Affiliations:** Department of Clinical Sciences, College of Veterinary Medicine, Qassim University, Buraydah 51452, Saudi Arabia; ahme.ali@qu.edu.sa

**Keywords:** male camel, reproductive emergencies, fertility, phimosis, paraphimosis

## Abstract

Reproductive emergencies in male dromedary camels (*Camelus dromedarius*) threaten fertility, health, and welfare but remain poorly documented. This review consolidates knowledge on the diagnosis, management, and prognosis of acute reproductive pathologies, including traumatic injuries (testicular trauma, penile hematoma, and preputial laceration), obstructive conditions (urethral obstruction, phimosis, and paraphimosis), vascular emergencies (spermatic cord torsion, priapism), inflammatory diseases (orchitis, epididymitis, pizzle rot), congenital anomalies (persistent frenulum), iatrogenic complications, and pharmacologic-induced dysfunction. Systematic breeding soundness examination and ultrasonography are indispensable for assessing testicular perfusion and guiding intervention. Given the scarcity of camel-specific literature, this narrative clinical review integrates evidence from published camelid studies, relevant reports from other domestic species, and representative clinical observations documented by the authors to provide a practical framework for diagnosis and management. Field constraints, the stoic nature of camels, and harmful ethnoveterinary practices often delay diagnosis and compromise outcomes. Prognosis varies considerably depending on the condition and timeliness of intervention; early aggressive management can preserve reproductive function, while delayed treatment frequently results in permanent infertility or death. Given the economic and cultural importance of camels in arid regions, improving outcomes for reproductive emergencies is essential for sustaining breeding programs and enhancing animal welfare. This narrative clinical review integrates published literature with representative clinical observations and retrospective field cases to summarize the diagnosis, management, and prognosis of reproductive emergencies in male dromedary camels. It also provides a structured clinical framework to support veterinarians in managing these acute conditions and highlights critical gaps requiring further research.

## Simple Summary

Male dromedary camels can suffer from sudden and serious reproductive problems that threaten their fertility, health, and even their lives. These emergencies include injuries to the testicles, penis, or prepuce, spermatic cord torsion, priapism, phimosis, paraphimosis, orchitis, pizzle rot, urethral obstruction, complications after castration, scrotal hernias, and problems caused by hormone or drug use. Many of these conditions are not well described in camels, and information is often borrowed from horses, cattle, or humans. Early diagnosis using clinical examination and ultrasound is critical. Treatment may include medications, surgery, or removal of the affected testicle or part of the penis. If treated quickly, some camels can recover and continue breeding. However, delayed treatment often leads to permanent infertility or death. Because camels are economically and culturally important in desert regions, veterinarians and camel owners must recognize these emergencies early and act promptly. This review provides a practical guide to help veterinarians diagnose and manage these urgent reproductive conditions in male camels.

## 1. Introduction

Reproductive emergencies in male dromedary camels (Table 1) represent a critical and often under-recognized domain of camelid medicine, posing significant threats to fertility, systemic health, and animal welfare. Unlike elective disorders, these emergencies demand immediate intervention to mitigate irreversible damage to reproductive structures and preserve breeding potential [1,2]. The economic and cultural importance of camels in arid regions further underscores the necessity of timely management. Infertility in male dromedaries arises from multiple causes requiring systematic investigation [3]. Despite their clinical significance, these emergencies remain poorly documented, with much knowledge extrapolated from equine, bovine, and small animal medicine. This gap highlights an urgent need for species-specific clinical guidelines tailored to the unique anatomical and physiological characteristics of the camel reproductive system [4,5,6].

Male camels are susceptible to a wide array of acute reproductive pathologies, categorized as traumatic, obstructive, inflammatory, vascular, and iatrogenic emergencies. Spermatic cord torsion, though rare, is a vascular catastrophe resulting from rotation of the spermatic cord, leading to ischemia and potential testicular loss if not corrected within hours, as reported in horses [7], humans [8,9], and camelids [4]. Testicular trauma and hematoma frequently occur due to bites or mounting injuries, presenting with acute scrotal swelling and possible rupture of the tunica albuginea [10]. While torsion of the appendix testis is more common in small animals, its occurrence in camels should not be dismissed.

The conditions reviewed in this article differ in their degree of urgency and clinical consequences. For clarity, they can be categorized into three groups: (i) true reproductive emergencies requiring immediate intervention to preserve life, welfare, or reproductive function (e.g., testicular torsion, priapism, severe penile or preputial trauma, and acute orchitis); (ii) urgent reproductive disorders that may rapidly compromise fertility, breeding ability, or animal welfare if left untreated (e.g., phimosis, paraphimosis, urethral obstruction, pizzle rot, and acute scrotal hernia); and (iii) breeding-soundness disorders with important reproductive implications but variable clinical urgency (e.g., persistent frenulum and reproductive dysfunction associated with performance-enhancing medications or hormonal treatments). This classification is intended to assist clinicians in prioritizing diagnosis and intervention according to the severity and immediacy of each condition (Table 2).

Vascular and hemorrhagic emergencies include spermatic cord hemorrhage, which may follow trauma or castration, and penile hematoma (“amputated penis”), resulting from rupture of the tunica albuginea during erection in horses and camels [11,12]. Such conditions can lead to significant blood loss and permanent impotence if not managed surgically. Priapism, a persistent painful erection unrelated to sexual stimulation, represents a neurovascular dysfunction requiring prompt intervention to prevent ischemic damage [2].

Obstructive urogenital emergencies are particularly prevalent, with urethral obstruction and urolithiasis (“water belly”) representing life-threatening conditions [6,13]. Calculi formation can lead to bladder rupture and death if not relieved. Paraphimosis, the inability to retract the protruded penis, is another common emergency resulting from trauma or edema and can progress to penile necrosis if not reduced promptly in humans and animals [14,15].

Traumatic injuries to the external genitalia are frequent in breeding camels. Penile trauma may include lacerations or fractures, while preputial lacerations often occur during copulation [9,12]. Acute scrotal hernia, though uncommon, can present as a painful scrotal enlargement requiring emergency herniorrhaphy. Inflammatory emergencies such as acute epididymitis and orchitis are typically bacterial, presenting with fever and scrotal swelling, and can lead to infertility if not treated aggressively [5].

Iatrogenic and management-related emergencies include post-castration complications such as hemorrhage and infection [9,10,16]. Pharmacologic emergencies can arise from improper use of reproductive hormones, resulting in prolonged anatomical and physiological dysfunction [17,18]. The diagnosis relies on clinical history, physical examination, and imaging such as ultrasonography, which are invaluable for assessing testicular perfusion and detecting hematomas [6,19,20,21,22]. However, field conditions and the camel’s stoic nature often complicate early intervention.

Because published information on reproductive emergencies in male dromedary camels remains scarce, the present article integrates evidence from peer-reviewed camelid literature with selected information from other domestic species when relevant. In addition, several sections include representative clinical observations and documented field cases encountered by the authors during routine veterinary practice. These observations are presented to illustrate clinical presentations and management approaches for conditions that remain poorly documented in the scientific literature and should not be interpreted as evidence equivalent to controlled clinical studies. It also consolidates current knowledge on the diagnosis and management of these emergencies, aiming to improve outcomes for affected animals worldwide.

## 2. Literature Search Strategy and Scope of the Review

The literature search was conducted using trustworthy search engines (PubMed, Google Scholar, etc.) and relevant veterinary textbooks to identify publications related to reproductive disorders and emergencies in male camelids. Search terms included combinations of consistent and appropriate keywords. The search primarily focused on publications available between 1980 and 2026.

Priority was given to peer-reviewed publications involving dromedary camels and other camelid species. However, because species-specific literature on male reproductive emergencies in camels remains scarce, relevant information from equine, bovine, small ruminant, and human medical literature was incorporated when it provided useful diagnostic, pathophysiological, or therapeutic insights applicable to camel practice. Additional information was derived from the authors’ documented clinical cases and more than two decades of clinical and research experience in camel reproduction. These observations were included primarily to illustrate rarely reported conditions and clinical presentations encountered in field practice.

To improve transparency, information presented throughout this review was categorized according to its source. Data derived from peer-reviewed publications are explicitly referenced, whereas observations originating from the authors’ documented clinical cases and long-term field experience are identified as clinical observations or field experiences. Such observations are included primarily to provide practical insights into conditions for which published camel-specific evidence remains limited.

The objective of this review was not to perform a systematic review or meta-analysis but rather to provide a practical, clinically oriented synthesis of the available evidence and expert experience. Consequently, formal systematic review methodologies, including PRISMA reporting, risk-of-bias assessment, and quantitative evidence synthesis, were not applied. Whenever possible, the strengths and limitations of the available evidence are highlighted throughout the manuscript.

The therapeutic recommendations presented are intended to provide general clinical guidance rather than prescriptive treatment protocols. Specific drug selection, dosage regimens, anesthetic techniques, surgical approaches, and postoperative management should be tailored to the individual animal, the severity of the condition, available facilities, and applicable veterinary regulations (Table 2).

## 3. Spermatic Cord Torsion

Testicular torsion, the rotation of the testis and spermatic cord causing vascular ischemia, is a critical urogenital emergency. While well described in humans, dogs, and horses, its occurrence in camelids is less defined but poses a significant threat to fertility in animals [6,23].

Clinical signs stem from acute vascular occlusion. In stallions, this manifests as colic, tachycardia, and unilateral scrotal swelling. A unique chronic case involved recurrent swelling over three years before infarction [7]. In camelids, classic torsion is rarely reported; acute scrotal swelling is more commonly linked to trauma, orchitis, or heat stress, mimicking torsion [10,24].

Diagnosis integrates physical exam and imaging. Palpation may reveal a firm, painful testis. Ultrasonography is essential for assessing parenchymal echotexture and, with Doppler, vascular flow [6,21]. Recent work [25] demonstrated that Doppler waveform alterations in the supratesticular artery correlate with semen biomarkers in infertile male dromedaries, providing a non-invasive prognostic tool. It differentiates torsion from hematomas or abscesses [26]. Exploratory surgery provides a definitive diagnosis [27].

Treatment is surgical. Orchiectomy is standard for necrotic or chronically compromised testes and is recommended for severe testicular pathology in camelids [1,24]. Orchiopexy is a salvage option only in acute, viable cases but is seldom advised. Prognosis depends on timing. Unilateral orchiectomy often yields good fertility if the contralateral testis is healthy [27]. However, an underlying anatomical predisposition can lead to contralateral torsion. In camelids, delayed treatment of scrotal swelling may risk permanent testicular degeneration [28].

## 4. Orchitis/Epididymitis

**Figure 1:**
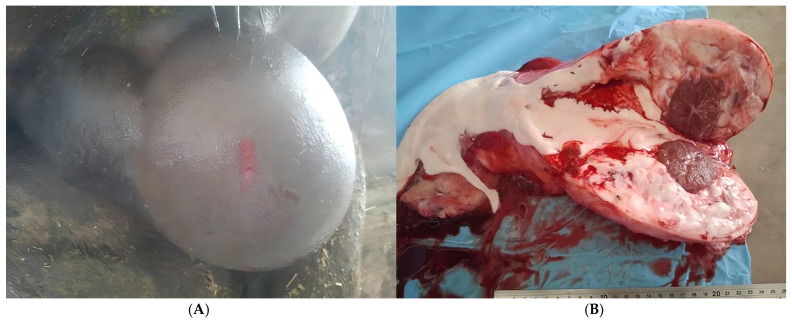
Acute orchitis (**A**) and testicular abscess (**B**) in dromedary camel.

Acute epididymitis and orchitis (Figure 1) are significant reproductive emergencies in the dromedary camel due to their rapid progression and potential to cause irreversible testicular damage. These conditions may arise from ascending infections, hematogenous spread, or traumatic injury during the rutting season. Infectious agents such as *Brucella* spp. have been implicated [5].

Clinically, affected animals present with the sudden onset of unilateral or bilateral scrotal swelling, increased testicular size, and marked pain. The scrotal skin is often warm and edematous. Behavioral changes include decreased libido and refusal to mate. Systemic signs such as fever and anorexia may accompany severe infections. If not promptly addressed, acute inflammation may progress to chronic degeneration [6].

Diagnosis combines clinical examination, ultrasonography, and laboratory testing. Ultrasonography allows visualization of parenchymal changes such as hypoechoic areas, fluid accumulation, and abscess formation [6]. Color Doppler ultrasonography can detect hyperemia associated with acute inflammation. Laboratory evaluation may reveal leukocytosis [5]. Differential diagnoses include testicular torsion, hematoma, and scrotal hernia [29].

Management requires immediate aggressive intervention. Broad-spectrum antimicrobial therapy should be initiated, ideally based on culture. Anti-inflammatory drugs are essential to reduce pain and secondary injury. Supportive management includes strict sexual rest. In cases with abscess formation, surgical drainage or unilateral orchiectomy may be required [24].

Prognosis depends on severity and duration. Early diagnosis and treatment are associated with a more favorable outcome, particularly in unilateral cases. Bilateral involvement often carries a guarded prognosis. Complications include testicular atrophy, fibrosis, and obstruction of sperm transport [5].

## 5. Testicular Trauma/Hematoma

**Figure 2:**
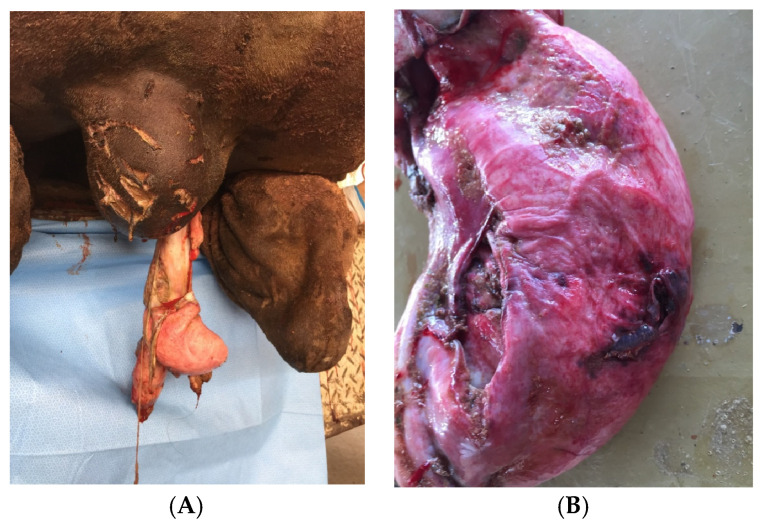
Fighting with other males during the rut season is frequently associated with testicular insults (**A**) or traumatic testicular avulsion (**B**).

Testicular trauma and associated hematoma formation (Figure 2) represent true reproductive emergencies in the dromedary camel due to the risk of irreversible testicular degeneration. These conditions most commonly occur during the rutting season from aggressive behavior, mounting attempts, or bites [15,29]. Affected camels present with acute unilateral or bilateral scrotal enlargement, pain on palpation, and reluctance to move. Local hyperthermia and edema are common. Chronic cases may present later with testicular atrophy or subfertility, particularly during the rutting season when the testes are highly vascularized in humans [30] and animals [27,31].

Diagnosis relies on clinical examination and advanced imaging. Palpation allows differentiation between edema and hematoma, though pain may limit assessment. Ultrasonography is the modality of choice, providing real-time evaluation of testicular parenchyma and tunica albuginea integrity [6,32]. Color Doppler ultrasonography is critical for assessing testicular perfusion, as compromised blood flow predicts a poor prognosis [27]. Differential diagnoses include orchitis, scrotal hernia, and neoplasia [5,6].

Immediate intervention aims to minimize hemorrhage and secondary damage. Conservative management for mild cases includes strict sexual rest, cold hydrotherapy, non-steroidal anti-inflammatory medications (NSAIDs), and antimicrobials [10]. Surgical intervention is warranted for progressive enlargement, tunica albuginea rupture, or compromised perfusion and may require partial or complete orchiectomy [11,24].

Prognosis depends on severity and rapidity of intervention. Mild unilateral hematomas may resolve with restoration of function. However, moderate to severe trauma frequently results in testicular fibrosis and permanent infertility, even unilaterally, due to thermoregulatory disruption and immune-mediated damage [27,31,33]. Complications include chronic orchitis, abscess formation, and reduced libido.

## 6. Penile Trauma

Penile trauma is an important reproductive emergency in male dromedary camels, particularly during the rutting season when aggressive interactions between competing males frequently result in genital injuries. Unlike in bulls, where rupture of the tunica albuginea with subsequent hematoma formation (“broken penis”) is more commonly reported, penile trauma in camels is more often characterized by lacerations caused by biting and fighting among rutting males [5,10,24]. These injuries may initially remain undetected because external swelling is often minimal, and affected animals may continue to exhibit normal libido. In many cases, the lesion is discovered only incidentally during attempted copulation when failure of penile protrusion or intromission becomes evident, often due to secondary phimosis caused by edema, fibrosis, a short amputated penis or cicatricial adhesion at the injury site [34,35]. Clinically, affected camels may present with impaired penile extension, pain during erection, unsuccessful mating attempts, and occasionally visible scar tissue or deformity.

**Figure 3:**
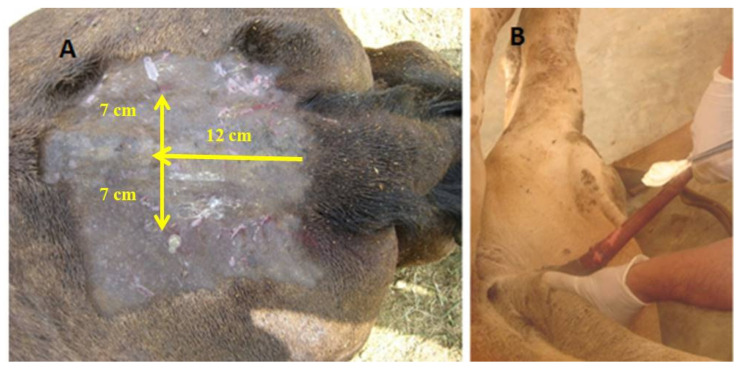
Pudendal nerve block in male dromedary camels carried out to ease the release of the penis from the prepuce according to Ahmed et al. (2011) [34]. (**A**) The site for anaethetic injection and (**B**) the manual extraction of the penis with blunt protective forceps for examination.

Diagnosis is based on clinical history and physical examination, supported by ultrasonography. A history of acute injury during fighting, with rapid penile and preputial hemorrhage, is highly suggestive. Pudendal nerve block [36] and extraction of the penis (Figure 3) using blunt long-handled forceps enable visualization of hematoma formation and disruption of the tunica albuginea. Differential diagnoses include preputial edema and urethral injury [11].

Management depends on severity. Mild cases may be managed conservatively with sexual rest and anti-inflammatory therapy, though this carries a risk of fibrosis. Surgical intervention is often recommended for moderate to severe cases, allowing evacuation of the hematoma and repair of the rupture site [11,24]. Males with amputated penis are advised to be discarded from breeding. Prognosis is highly dependent on the timing of intervention; early diagnosis and surgical management are associated with a more favorable outcome.

## 7. Priapism

Priapism, defined as a persistent and often painful penile erection lasting more than four hours in the absence of sexual stimulation, constitutes a true urological emergency in male dromedary camels. This condition results from dysregulation of normal detumescence and, if not promptly managed, leads to ischemic injury, penile fibrosis, and permanent loss of breeding ability [37,38]. In camels, priapism is rarely reported, but based on the authors’ experience, it occurs most frequently during the rutting season and is often associated with prolonged sexual excitement, neurologic dysfunction, or administration of pharmacologic agents [2].

Clinically, affected camels present with a fully erect, non-retractable penis that remains protruded for an extended duration. The glans and penile shaft appear engorged, firm, and often cyanotic. Animals may show distress, including restlessness, straining, and reluctance to move. In advanced cases, the penis becomes cold and darkened, indicating impending necrosis [2].

The etiology is classified into low-flow (ischemic) and high-flow (non-ischemic) priapism. Low-flow priapism, the more common and dangerous form, results from venous outflow obstruction leading to hypoxia and necrosis. It may be triggered by prolonged mating, spinal cord lesions, or sedatives such as acepromazine or xylazine [37,39]. High-flow priapism, caused by unregulated arterial inflow due to penile trauma, is less common but should be considered following injury [40,41].

Diagnosis is based on clinical presentation and penile examination. Differentiation between low-flow and high-flow is critical. In low-flow priapism, the penis is rigid, painful, and dark, with absent blood flow on Doppler ultrasonography. In high-flow priapism, the penis is partially tumescent, often painless, with normal or increased flow [21,42]. Ultrasonography with color Doppler is the diagnostic modality of choice [6,32]. Blood gas analysis of cavernosal aspirate confirms ischemia in low-flow priapism [38].

Management requires immediate intervention. In low-flow priapism, initial treatment focuses on pain relief, sedation, and attempted manual reduction. If unsuccessful, intracavernosal aspiration followed by irrigation with dilute phenylephrine may be attempted [37,38]. If medical therapy fails or necrosis is evident, surgical intervention is required, including cavernosal–spongiosal shunts or partial penectomy [24,43]. In high-flow priapism, conservative management may suffice, but persistent cases may require arterial embolization [40,41].

Complications are frequent and severe. Prolonged ischemia leads to cavernosal fibrosis, erectile dysfunction, and permanent loss of breeding capability [38,44]. Recurrent priapism (stuttering priapism) has been described in humans and may occur in camels [9]. Prognosis is guarded to poor, particularly in low-flow cases with delayed treatment beyond 12–24 h [2]. Early recognition offers the best chance of preserving function. Priapism must be regarded as a true reproductive emergency in the dromedary camel.

## 8. Persistent Frenulum

**Figure 4:**
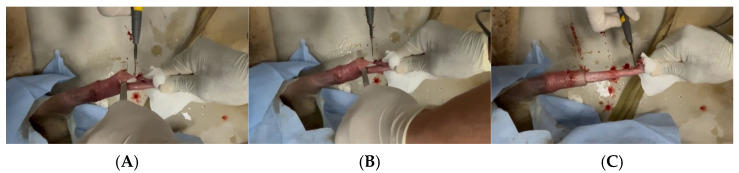
Persistent frenulum of the penis in 5 year-old male dromedary camel affected with phimosis just before thermo-cauterization (**A**), during surgery (**B**) and after surgery (**C**).

Persistent penile frenulum (Figure 4) is a congenital developmental anomaly characterized by failure of complete separation between the penis and prepuce after puberty. Consequently, affected males are commonly discouraged from breeding, and castration has been recommended in some species to prevent transmission of the defect to future generations [42]. However, in dromedary camels, the importance of this condition is not limited to its potential genetic implications. During the early breeding life of affected males, the persistent frenulum interferes with normal penile extension and intromission, resulting in repeated mating failure, animal frustration, and reduced reproductive efficiency [15,27]. Therefore, this condition should be considered a reproductive emergency that warrants timely surgical intervention to restore normal copulatory function and breeding performance [12]. While recognized in other species, it has not previously been documented in dromedary camels. The authors encountered two mature male camels with repeated unsuccessful mating attempts, failed penile protrusion, and sexual frustration. Both exhibited clinical signs consistent with phimosis, including incomplete extrusion and ventral deviation, raising concerns for breeding soundness and welfare.

Diagnosis is primarily clinical and relies on careful penile examination under controlled protrusion. Manual extrusion revealed a distinct fibrous band restricting full glans extension, paralleling descriptions in cattle and small animals [42,45,46]. Differentiation from true phimosis and inflammatory balanoposthitis is essential [47]. The congenital nature of the condition and its characteristic appearance support the diagnosis.

Surgical intervention is definitive. In the two camel cases, correction was performed using thermo-cauterization under analgesia. Following penile extrusion, the frenular band was transected, allowing immediate release. Thermo-cauterization minimized hemorrhage [48]. Postoperative management included sexual rest, local wound care, and anti-inflammatory therapy. Both animals showed uncomplicated healing.

## 9. Paraphimosis

**Figure 5:**
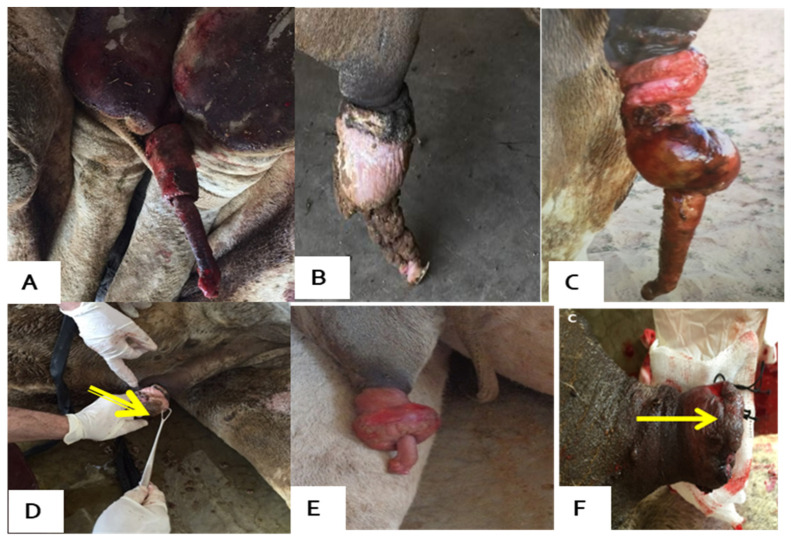
Paraphimosis in camel may be admitted at varying durations after occurrence relatively fresh (**A**), relatively old (**B**) and old (**C**). Handling old Paraphimosis with curetting (arrow) (**D**), cold douches and cleaning (**E**) and placement of a purse-string suture (arrow) to prevent recurrence (**F**).

Paraphimosis (Figure 5) is a true urological emergency characterized by the inability to retract the protruded penis into the preputial sheath, with the prepuce forming a constricting ring behind the glans [14,15,49]. In camelids, this presents as an acute, painful state with signs of distress including colic, restlessness, and a stiff gait [10]. The hallmark is a visibly prolapsed, swollen, and edematous penis that appears dark red to purple due to venous congestion [24,34]. Common etiologies include traumatic injuries during copulation or fighting, complications of sand masturbation, iatrogenic causes, and strangulation from preputial rings [4]. As the condition progresses, ischemia can lead to coldness and black discoloration of the glans, indicating impending necrosis. The condition worsens with neglect and delayed intervention.

Diagnosis is immediate based on the acute clinical presentation and history. Direct examination confirms the prolapsed state and degree of vascular compromise [10]. Differentiating between viable and non-viable penile tissue is critical for determining intervention.

Management is an acute emergency focused on reducing edema and replacing the organ before ischemic damage occurs. Initial first aid involves protecting the exposed penis with saline-soaked bandages [1]. Definitive treatment requires sedation and analgesia. Manual reduction is preceded by efforts to reduce severe edema using osmotic agents like granulated sugar or hypertonic solution-soaked gauze [24]. Surgical intervention is necessary if manual reduction fails or if necrosis is present. An emergency dorsal slit relieves pressure [15], and if gangrene is evident, partial penectomy is required. To prevent recurrence, placement of a purse-string suture while maintaining an adequate opening for urine passage is essential during the procedure. Sexual excitation without going through the mounting process is crucial to prevent expected penile–preputial adhesions.

Prognosis is guarded to poor and directly correlated with the duration prior to treatment. Prompt reduction offers the best chance of recovery, though some fibrosis is common. Delaying treatment leading to necrosis necessitates penectomy, resulting in permanent infertility [10].

## 10. Phimosis

**Figure 6:**
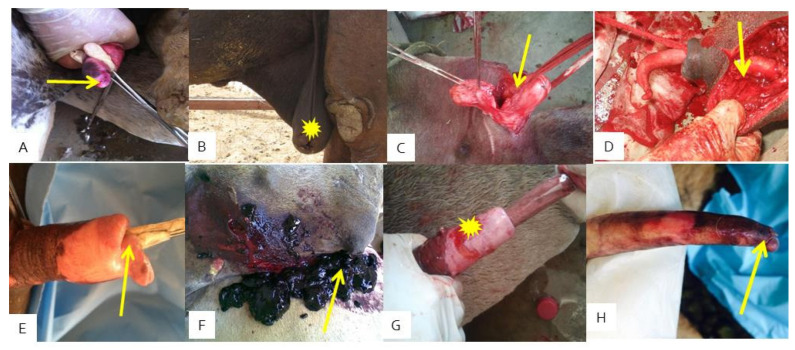
Phimosis in camels associated with different penile and preputial affections ranging from amputated (arrow) penis (**A**), posthitis (star) (**B**), penile fibroma (arrow) (**C**), penile adhesion (arrow) along the shaft of the penis (**D**), adhesions (arrow) at the glans (**E**), penile trauma (arrow) and hematoma (**F**), preputial lacerations (star) (**G**), and a scalded (arrow) penis (**H**).

Phimosis (Figure 6), the inability to protrude the penis from the prepuce, is a significant reproductive disorder in male camelids that directly impairs breeding ability (impotentia coeundi). In dromedary camels, this condition manifests as failure to achieve intromission during the rutting season, leading to observable infertility [5,35,47,49].

A critical distinction is between congenital and acquired forms. While congenital phimosis from preputial stenosis is noted, the majority of cases are acquired secondary to preputial or penile pathology [12,35,47]. Affected animals typically have a prior history of successful breeding, with the condition arising following trauma or inflammation from aggressive behaviors such as fighting [1,10,24,34]. In some cases, adhesions or strictures following injury can also lead to a non-retractable prepuce [35]. Affected males may show signs of discomfort or dysuria, particularly if complicated by urinary calculi. Erectile dysfunction is a common form of phimosis in camels that directly compromises breeding ability and may result in substantial economic losses during the limited breeding season. Similar impotentia generandi patterns in male dromedaries correlate clinical findings with semen characteristics and testicular histopathology [19,50]. Affected camels exhibit an inability to achieve or maintain penile erection, often accompanied by altered serum concentrations of nitric oxide metabolites, cardiac troponin I, and testosterone, indicating underlying vascular, muscular, and endocrine disturbances [51].

Diagnosis is primarily clinical, anchored in a systematic breeding soundness examination and history [6]. The definitive step involves careful exteriorization of the penis under sedation or pudendal nerve block [26] to identify obstructive lesions. Supportive diagnostics include hematology revealing inflammation [35,51], and elevated serum nitric oxide metabolites (NOMs) as a marker of tissue damage. Using computer-assisted sperm analysis, epididymal sperm quality in camels with penile and preputial pathologies showed significant impairments in sperm motility parameters [12]. Testosterone concentrations typically remain normal, indicating an anatomic or inflammatory rather than an endocrine etiology [35]. In complex cases, histopathological examination is definitive [52].

Management is dictated by the underlying cause. Initial medical management for active infection employs systemic antibiotics and NSAIDs alongside topical wound care [10,24]. Sexual rest is mandatory. However, surgical intervention is frequently required for chronic fibrosis or adhesions [10]. Options include preputioplasty or circumcision (posthetomy), with resection of obstructive masses as needed.

Prognosis for future breeding soundness is guarded to fair and highly dependent on early diagnosis and successful surgical intervention [1,34]. If untreated, the condition leads to permanent infertility. Chronic cases risk progression to paraphimosis, ascending urinary tract infections, and secondary testicular degeneration [35].

## 11. Preputial Laceration/Prolapse

**Figure 7:**
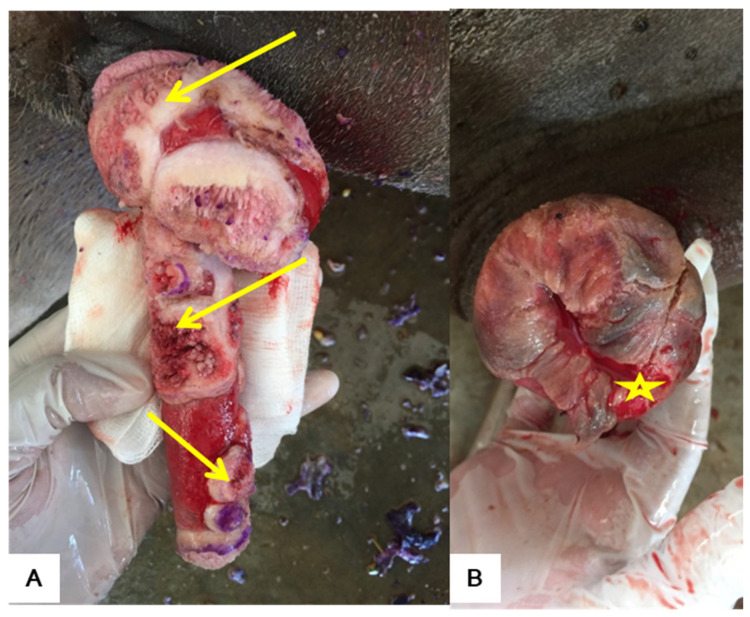
Preputial and penile necrotic lesions after curation and washing (arrows) (**A**) and preputial laceration (star) and insults (**B**) in a camel.

Preputial laceration and prolapse (Figure 7) are important reproductive emergencies in male dromedary camels, particularly during the rutting season. These conditions arise from forced intromission, accidental injuries, or improper handling. Clinically, affected camels present with preputial swelling, protrusion of the mucosa, and visible lacerations [10,24].

In prolapse cases, the preputial lining becomes edematous, congested, and susceptible to contamination. Lacerations are accompanied by hemorrhage and pain. Secondary signs include dysuria and reduced libido. Severe cases may lead to necrosis and systemic signs due to secondary infection. Chronic cases may present with fibrosis and impaired penile protrusion [15,29].

Diagnosis is based on clinical examination and direct visualization under adequate restraint. Ultrasonography may be useful for assessing deeper tissue involvement. Differential diagnoses include balanoposthitis and urethral obstruction [11]. Management requires prompt intervention. Initial treatment focuses on reducing inflammation, controlling hemorrhage, and protecting exposed tissues. Simple prolapse may be managed with manual reduction and retention sutures. Moderate to severe lacerations require surgical debridement and reconstruction [24].

Prognosis depends on severity and timeliness of intervention. Early management of mild to moderate cases is associated with favorable outcomes, while severe or neglected cases often result in permanent structural damage and impaired fertility.

## 12. Urethral Obstruction/Calculi (“Water Belly”)

**Figure 8:**
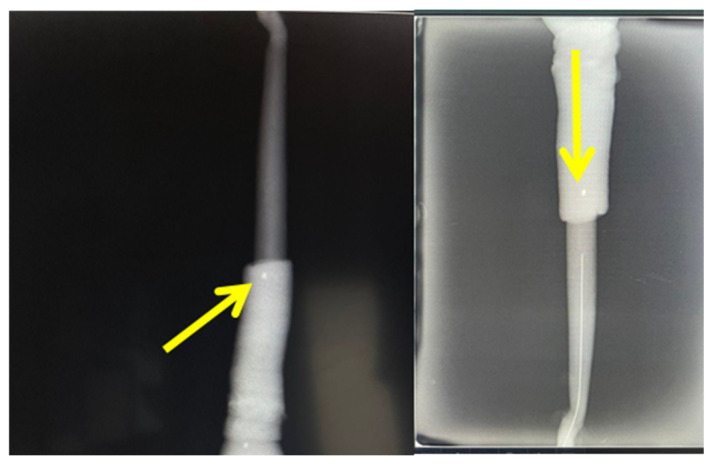
Urine retention due to urolithiasis (arrows) is not uncommon in male camels.

Urethral obstruction (Figure 8) in the dromedary camel constitutes a true reproductive and medical emergency due to rapid progression toward metabolic derangements, bladder rupture, and death. Clinically, affected males present with progressive dysuria, stranguria, and signs of abdominal discomfort. As obstruction persists, animals develop restlessness, bruxism, and dehydration. In advanced cases, rupture of the urethra or bladder leads to urine extravasation and ventral abdominal edema (“water belly”) [53]. A considerable proportion of cases are associated with penile ulceration and strictures following mating, particularly in camels treated with ethnoveterinary caustic substances.

Diagnosis is based on clinical findings, ultrasonographic evaluation, and laboratory assessment. Ultrasonography and radiography are indispensable, allowing detailed assessment of the urinary bladder, kidneys, and urethra, as well as detection of uroliths and urine leakage [53,54]. Sonographic findings may include hyperechoic calculi with acoustic shadowing and hydronephrosis [13,55]. Laboratory investigations reveal azotemia and evidence of oxidative stress [56]. Differential diagnoses include urethral rupture and cystitis [57].

Management requires immediate intervention to relieve urinary outflow. Initial care includes analgesia, anti-inflammatory therapy, and antimicrobials. Urethral catheterization is often challenging, though recent advances have demonstrated novel techniques [53,58]. When catheterization fails, tube cystostomy has been successfully employed as a lifesaving procedure [53,59]. Long-term management focuses on dietary modification and treatment of concurrent penile lesions.

Prognosis depends on the duration of obstruction. Early detected cases have a favorable outcome, whereas delayed presentation is associated with high morbidity and mortality. Complications include urethral rupture, uroperitoneum, hydronephrosis, and chronic urethral strictures [53,56].

## 13. Post-Castration Emergencies

Castration in the dromedary camel may be associated with acute postoperative complications that can progress into life-threatening emergencies. Clinical presentation includes hemorrhage, scrotal swelling, and systemic signs of distress [60]. Acute hemorrhage may present as continuous bleeding from the surgical site, leading to hypovolemic shock [10].

Scrotal edema frequently develops and may be accompanied by local heat and pain. In cases complicated by infection, animals may exhibit fever and purulent discharge. More severe presentations include evisceration and septic funiculitis [1,10,24,34]. Diagnosis is based on clinical examination. Ultrasonography provides information regarding fluid accumulation, hematoma, or abscess formation. Differential diagnoses include inguinal hernia [11].

Management requires rapid targeted intervention. Hemorrhage should be addressed through ligation of the spermatic cord. Scrotal edema can be managed conservatively with anti-inflammatory drugs and cold hydrotherapy. Evisceration constitutes a surgical emergency requiring immediate correction [24].

Complications include hemorrhagic shock, severe infection, and evisceration, all with high mortality risk if not treated promptly. Prognosis depends on the type and severity of the complication and the timeliness of intervention.

## 14. Scrotal Hernia

The male dromedary camel possesses distinctive reproductive anatomical features that influence scrotal hernia presentation. Unlike other livestock, the camel scrotum is non-pendulous and situated high in the perineal region, with the testes directed caudo-dorsally [28]. Acute scrotal hernia occurs when abdominal contents protrude through the inguinal canal into the scrotal cavity, potentially compromising testicular soundness.

Male camels with acute scrotal hernia typically present with sudden-onset scrotal enlargement, often accompanied by abdominal pain. The clinical history frequently reveals recent physical exertion or straining. On examination, the affected hemiscrotum is asymmetrically enlarged. Palpation may reveal a soft, reducible mass in omental herniation, whereas incarcerated intestinal loops are firm and non-reducible. The non-pendulous scrotum can initially obscure the diagnosis [10,61].

Accurate diagnosis requires clinical evaluation complemented by imaging. Ultrasonography is particularly valuable for differential diagnosis [6,61], revealing hyperechoic omental or intestinal segments adjacent to the testicular parenchyma. Doppler ultrasonography can assess testicular viability. Differential diagnoses include hydrocele and orchitis [6].

Prompt surgical intervention is indicated, especially when strangulation is suspected. Preoperative stabilization includes fluid therapy. Surgical management involves herniorrhaphy via an inguinal approach. If testicular viability is compromised, unilateral orchiectomy may be necessary. Postoperative management includes antimicrobials and restricted activity.

Prognosis depends on the duration of herniation and testicular viability at surgery. Cases with intact perfusion carry a favorable prognosis for return to breeding soundness.

## 15. Persistent Genital Arousal Disorder

To the authors’ knowledge, this condition has not previously been described in veterinary species. The following description is based primarily on a documented clinical case encountered by the authors and is supplemented by information available from the human medical literature.

Persistent genital arousal disorder (PGAD) is a rare condition characterized by spontaneous, intrusive, and persistent genital arousal in the absence of sexual desire. While documented in human medicine as a sexual health disorder with significant implications [62,63,64], its occurrence in veterinary species has remained largely unreported. In this report, the authors present the first documented case, covering presentation, diagnosis, attempted management, and prognosis in a male dromedary camel.

The clinical manifestation in the reported male dromedary was acute and progressive. The subject developed symptoms abruptly after mating with an estrous female treated with some sort of ethnoveterinary prescription. Primary signs included continuous pelvic thrusting, persistent penile erection, and repeated attempts to ejaculate without external stimuli. This was compounded by systemic illness: recumbence, anorexia, hyperthermia, dehydration, and intense muscular seizures. This presentation aligns with human PGAD descriptions, though the systemic collapse appeared more extreme [65,66].

Diagnosis was primarily based on distinctive clinical signs, as no confirmatory test exists in animals. A comprehensive breeding soundness examination, including scrotal palpation and ultrasonography, revealed no structural abnormalities [6,64]. Hematology showed dehydration and leukocytosis indicative of systemic inflammation [35,64]. Biochemistry revealed elevated creatinine (acute renal dysfunction) and amylase (possible pancreatic involvement), highlighting severe multi-organ sequelae.

Management was symptomatic and supportive. Initial emergency treatment with flunixin meglumine failed. The therapeutic plan included aggressive fluid therapy, analgesics, and muscle relaxants. Unfortunately, the animal died during treatment, underscoring the refractory nature of the condition. Based on this single case, the prognosis for advanced PGAD appears grave. The relentless muscular activity led to exhaustion, hyperthermia, and likely rhabdomyolysis, contributing to acute kidney injury [65]. The exact cause of death remained unresolved as the owner disapproved necropsy.

## 16. Pizzle Rot

**Figure 9:**
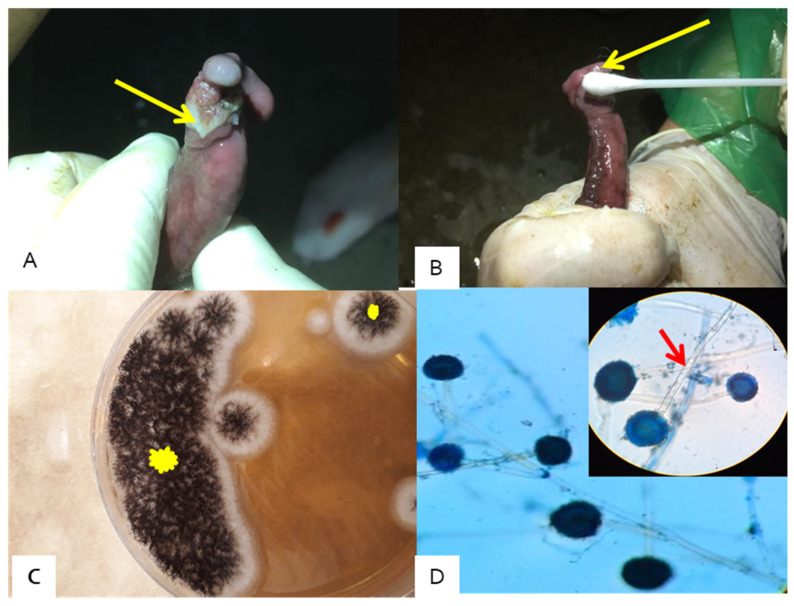
Pizzle rot in a male dromedary camel. (**A**) Ulcerative and necrotic lesions (arrow) affecting the glans penis following prolonged inflammation; (**B**) collection of microbiological samples from ulcerative lesions using sterile swabs (arrow); (**C**) black fungal colonies isolated from lesion samples following culture (stars); and (**D**) microscopic appearance of fungal hyphae (arrow) consistent with *Aspergillus niger*.

Pizzle rot (Figure 9) is classically described in cattle as a necrotizing infection of the distal penis and prepuce; however, to the authors’ knowledge, this condition has not previously been reported in dromedary camels. Based on clinical observations and closely related reported cases [67], pizzle rot should be recognized as a severe reproductive emergency in this species. Based on the authors’ clinical observations, affected camel bulls may present during the rutting season with acute penile swelling, foul-smelling discharge, hemorrhage, and inability to protrude the penis. The condition is frequently preceded by mating-related trauma and aggravated by inappropriate ethno-veterinary interventions, including topical caustic substances.

Diagnosis is primarily clinical but requires full exteriorization of the penis under sedation. Gross findings of necrotizing balanoposthitis are highly suggestive. Microbiological confirmation is essential, as mixed infections appear central. In the representative case illustrated in Figure 9, samples were collected from ulcerative penile lesions using sterile swabs and submitted for microbiological examination. Samples were cultured on Sabouraud dextrose agar and incubated under standard laboratory conditions. Colony morphology was evaluated macroscopically, and fungal elements were examined microscopically following routine staining procedures. The presence of *Aspergillus* spp. and Candida albicans was confirmed based on characteristic colony appearance and microscopic morphology. These findings supported the diagnosis of mycotic balanoposthitis associated with pizzle rot [68,69,70]. Differential diagnoses include traumatic balanitis and urethral obstruction.

Pizzle rot requires immediate aggressive intervention. Initial management focuses on analgesia, anti-inflammatory therapy, topical antimycotics, and local wound care. Necrotic tissue should be debrided, and systemic antifungal therapy instituted when mycotic infection is confirmed [71]. Sexual rest is mandatory. Owner education is critical, as continued application of caustic ethnoveterinary remedies worsens lesions.

Complications include progressive penile necrosis, deep fibrosis, phimosis, and complete loss of breeding ability. Prognosis depends on the stage at presentation. Early, aggressively treated cases may recover partial function, whereas advanced necrotic cases carry a guarded prognosis.

Prognosis following surgical correction is excellent, with most animals returning to normal sexual function [45,48]. Given the potential hereditary component suggested in other species, affected animals should be monitored closely.

## 17. Performance-Enhancing Medications and Hormonal Treatments

The use of performance-enhancing medications, including anabolic-androgenic steroids and exogenous testosterone, is increasingly recognized as a contributor to reproductive dysfunction in male dromedary camels. Clinically, affected animals often present with reduced libido and decreased breeding efficiency. In some cases, transient hypersexual behavior may be observed, followed by a decline in sexual performance [15,29,72].

Testicular changes include reduced size, softening, and impaired spermatogenesis. Field observations indicate that racing camels treated with anabolic steroids frequently exhibit testicular degeneration and subfertility [73,74]. Semen abnormalities include decreased sperm motility and increased morphological defects. Behavioral alterations such as aggression may accompany endocrine disruption [75].

Diagnosis relies on clinical history, reproductive examination, and laboratory evaluation. Breeding soundness examination reveals abnormalities in testicular consistency and semen quality. Endocrine profiling plays a central role. Administration of exogenous androgens suppresses the hypothalamic–pituitary–gonadal axis, resulting in decreased LH and FSH [17]. Affected camels may exhibit low endogenous gonadotropins despite normal or elevated testosterone [18]. Semen evaluation often reveals reduced motility, consistent with endocrine-mediated disruption of spermatogenesis.

Management is primarily based on cessation of the offending agent. Discontinuation of anabolic steroids is essential. Gradual recovery of the axis may occur over weeks to months [17]. Supportive management includes optimizing nutrition and reducing stressors. Hormonal therapy to restore gonadotropin secretion may be considered, though its efficacy in camels is undocumented.

Complications are significant. Chronic suppression leads to testicular atrophy and long-term infertility. Histological changes include degeneration of the seminiferous tubules [40,41]. Prognosis is guarded, especially in heavily treated animals. Early recognition and preventive education are essential to preserve reproductive efficiency.

The quantity and quality of evidence available for reproductive emergencies in male dromedary camels (Table 3) vary considerably among conditions. While disorders such as orchitis, epididymitis, phimosis, paraphimosis, and urethral obstruction are supported by published camel-specific studies and clinical reports, several other conditions rely primarily on isolated case reports, extrapolation from other domestic species, or the authors’ documented clinical observations. To improve transparency, Table 3 summarizes the evidence supporting the diagnosis, treatment, and prognosis for each condition and highlights areas where additional camel-specific research is needed.

This review is inherently limited by the profound scarcity of species-specific literature on reproductive emergencies in male dromedary camels. Despite the economic and cultural importance of camels in arid regions [3,76], most current knowledge is extrapolated from other domestic species or derived from isolated case reports and the authors’ long-standing clinical experience. Field constraints—including limited veterinary infrastructure, the stoic nature of camels, which masks early clinical signs, harmful ethnoveterinary practices that complicate presentations, and cultural prohibitions against necropsy—severely hamper data collection and research progress [35,60]. Fundamental gaps exist for nearly every condition reviewed: the true prevalence and risk factors are unknown; standardized diagnostic protocols are lacking, particularly for poorly documented conditions such as persistent genital arousal disorder and pizzle rot; optimal medical and surgical management strategies have not been validated through controlled trials; and long-term reproductive outcomes following intervention remain unevaluated [19,77]. The potential roles of infectious agents, including trypanosomiasis and mycotic pathogens, in precipitating reproductive emergencies require further investigation [67]. Future research must prioritize prospective multicenter studies, advanced imaging research including standardized Doppler ultrasonographic evaluation of testicular perfusion [25], and educational initiatives to discourage harmful traditional practices. Only through such efforts can an evidence-based framework be established to improve diagnostic accuracy, guide effective management, and preserve the reproductive sustainability of this invaluable species.

An additional limitation of this review is that, for several conditions, camel-specific evidence is restricted to isolated case reports or the authors’ documented clinical observations. Consequently, some diagnostic and therapeutic recommendations are based on expert clinical experience and extrapolation from related species rather than on controlled studies in dromedary camels. These recommendations should therefore be interpreted as practical clinical guidance rather than definitive evidence-based protocols.

Not all conditions discussed in this review constitute classical surgical emergencies. Some, such as persistent frenulum and drug-induced reproductive dysfunction, are primarily breeding-soundness disorders, whereas others, including testicular torsion, priapism, severe penile trauma, and post-castration complications, require immediate intervention. Recognition of these distinctions is essential for appropriate clinical prioritization and management.

## 18. Conclusions

Male reproductive emergencies in dromedary camels represent a diverse and often life-threatening group of conditions that demand immediate recognition and intervention. From traumatic, obstructive, and vascular pathologies to inflammatory and iatrogenic emergencies, these conditions pose significant threats to fertility and animal welfare. The unique anatomical characteristics of the camel necessitate a tailored clinical approach. By consolidating current knowledge and highlighting gaps, this review underscores the urgent need for a structured, evidence-based framework to improve diagnostic accuracy, guide effective management, preserve valuable genetic resources, and safeguard the reproductive sustainability of camels.

## Figures and Tables

**Table 1:** Reproductive Emergencies in Male Dromedary Camels.

Affected Organ/System	Emergency Condition	Main Clinical Signs	Diagnostic Tools	Possible Consequences	Main Management	Evidence Source	Evidence Level
Testis and Epididymis	Testicular torsion	Acute scrotal pain, swelling, sudden onset	Ultrasonography, palpation	Ischemia, rapid necrosis, infertility	Emergency orchiectomy/detorsion	Camelid reports, equine and bovine literature	Limited camel-specific evidence + extrapolation
	Orchitis/Epididymitis (Acute)	Fever, painful scrotal enlargement, lameness	US, CBC, culture	Testicular degeneration, infertility	Antibiotics, anti-inflammatory; unilateral castration if severe	Camel clinical studies, breeding soundness investigations	Moderate camel-specific evidence
	Testicular Trauma/Hematoma	Scrotal enlargement, hemorrhage, pain, potential rupture	Ultrasonography, clinical exam	Testicular rupture, degeneration, atrophy	Conservative management (cold packs, NSAIDs) or surgery/drainage	Camel clinical reports, reproductive case series, comparative veterinary literature	Moderate camel-specific evidence
Penis	Penile Trauma/amputated/hematoma (Broken penis)	Sudden swelling, bruising, inability to breed after mating	Clinical exam, US	Fibrosis, hematoma, impotence	Surgical repair; medical management for small hematomas	Camel case reports, bovine and equine literature	Limited camel-specific evidence + extrapolation
	Priapism	Persistent erection unrelieved by ejaculation	Clinical exam	Ischemic necrosis, fibrosis	Sedation, analgesics; surgical shunting if prolonged	Published camel case reports, human and equine literature	Moderate camel-specific evidence
	Persistent frenulum	Deviation of penis during copulation, inability to intromit	Breeding soundness exam	Breeding failure	Surgical correction (frenulotomy)	Authors’ documented clinical cases, canine and feline case reports, bovine reproductive literature	Primarily clinical observations + extrapolation
Prepuce	Paraphimosis	Inability to retract penis into sheath; protrusion	Clinical exam	Necrosis, drying, trauma of glans	Cleaning, lubricants, reduction; surgery if severe	Camel case reports, camelid literature, equine literature	Moderate camel-specific evidence
	Phimosis	Inability to protrude penis from sheath	Clinical exam	Infertility (failure to breed)	Surgical correction of preputial orifice	Published camel clinical studies and case series	Strong camel-specific evidence
	Preputial Laceration/Prolapse	Edema, hemorrhage, visible tissue damage	Clinical examination	Adhesions, strictures, breeding failure	Surgical repair, debridement, antibiotics	Camel clinical reports and case observations	Limited camel-specific evidence
	Pizzle rot (Ulcerative Posthitis)	Necrotic, ulcerative scabs at preputial opening; malodor, dysuria	Clinical exam, ruling out urolithiasis	Urethral stricture, phimosis, septicemia	Dietary change (reduce protein), topical antibiotics, debridement	Authors’ documented clinical cases, fungal culture findings, bovine literature	Clinical observations + limited comparative evidence
Urethra and Urinary Tract	Urethral Obstruction/Calculi (“Water belly”)	Stranguria, anuria, abdominal pain, ventral edema	US, catheterization, chemistry (azotemia)	Bladder rupture, uremia, death	Emergency urethrostomy, relief of obstruction, fluid therapy	Camel clinical reports, ultrasonographic studies, surgical case reports	Moderate camel-specific evidence
Spermatic Cord/Vascular	Post-Castration Emergencies	Hemorrhage (shock, bleeding), eventration (tissue protruding)	Clinical evaluation	Hemorrhagic shock, peritonitis, death	Ligation of vessels, hernia repair, transfusion, antibiotics	Camelid literature, camel clinical observations, reproductive surgery references	Limited camel-specific evidence
Inguinal Region	Acute Scrotal Hernia	Enlarged, painful, non-reducible scrotum	US, palpation	Strangulation of intestine	Emergency herniorrhaphy	Camel case reports and comparative livestock literature	Limited camel-specific evidence + extrapolation
Neurologic/Behavioral	Persistent genital arousal disorder	Persistent sensation of genital arousal without desire; restlessness	History, behavioral assessment, rule-out organic causes	Exhaustion, self-trauma, infertility	Environmental management, analgesics, behavioral modification	Authors’ documented clinical case, human medical literature	Single clinical observation + extrapolation
Iatrogenic/Pharmacologic	Performance-Enhancing Medications and Hormonal Treatments	Abnormal libido (excessive or suppressed), erectile dysfunction, testicular atrophy	History of drug administration, hormone assay	Reproductive dysfunction, infertility	Drug withdrawal; supportive therapy; GnRH/hCG for hormonal stimulation	Camel reproductive studies, endocrinology literature, human infertility literature	Moderate camel-specific evidence + extrapolation

Evidence classification: Strong camel-specific evidence = supported by multiple camel studies or case series; Moderate camel-specific evidence = supported by limited camel studies, case reports, or clinical investigations; Limited camel-specific evidence = primarily supported by isolated camel reports and extrapolation from other species; Clinical observations = based predominantly on the authors’ documented field cases and clinical experience supplemented by comparative literature.

**Table 2:** Clinical classification and emergency stabilization of the reproductive emergencies in male dromedary camels.

Condition	Clinical Classification	Emergency Stabilization	Definitive Treatment
Testicular trauma	True reproductive emergency	Analgesia, anti-inflammatory therapy, stabilization of systemic injuries, ultrasonographic evaluation	Conservative management for minor injuries; surgical exploration or orchiectomy for severe trauma or non-viable testes
Testicular torsion	True reproductive emergency	Immediate analgesia, fluid therapy if indicated, assessment of systemic status	Emergency surgical exploration with detorsion or orchiectomy depending on testicular viability
Orchitis/Epididymitis (acute)	True reproductive emergency	Analgesia, anti-inflammatory therapy, antimicrobial treatment when infection is suspected	Targeted antimicrobial therapy, supportive care, unilateral orchiectomy in severe or chronic irreversible cases
Penile hematoma	True reproductive emergency	Analgesia, restriction of breeding activity, control of inflammation	Conservative management in mild cases or surgical repair of tunica albuginea rupture in severe cases
Preputial laceration	True reproductive emergency	Hemorrhage control, wound cleansing, analgesia, antimicrobial coverage	Surgical reconstruction, debridement, and postoperative wound management
Paraphimosis	True reproductive emergency	Reduction in edema, lubrication, protection of exposed penile tissues, analgesia	Manual reduction, preputial reconstruction, or surgical correction when necessary
Priapism	True reproductive emergency	Analgesia, anti-inflammatory therapy, assessment of tissue viability and hydration status	Medical management in early cases; surgical intervention in persistent or irreversible cases
Urethral obstruction	Urgent reproductive disorder with systemic consequences	Correction of dehydration and electrolyte imbalances, pain management, urinary decompression when possible	Urethral catheterization, tube cystostomy, urethrostomy, or other surgical procedures according to lesion severity
Post-castration emergencies	True reproductive emergency	Control of hemorrhage, fluid therapy, analgesia, management of shock when present	Ligation of bleeding vessels, surgical correction of evisceration, drainage or treatment of infection as indicated
Acute scrotal hernia	Urgent reproductive disorder	Patient stabilization, fluid therapy, pain management, assessment of intestinal viability	Herniorrhaphy with reduction in herniated contents; orchiectomy if testicular viability is compromised
Phimosis	Urgent reproductive disorder	Control of inflammation and secondary infection, analgesia	Surgical correction of preputial stenosis and restoration of penile protrusion
Pizzle rot	Urgent reproductive disorder	Analgesia, wound cleansing, control of infection, prevention of further trauma	Debridement of necrotic tissue, antimicrobial/antifungal therapy, and long-term wound management
Persistent frenulum	Breeding-soundness disorder with reproductive implications	No specific emergency stabilization usually required; breeding restriction	Surgical transection of the persistent frenulum and postoperative sexual rest
Performance-enhancing medications/hormonal treatments	Breeding-soundness disorder with reproductive implications	Discontinuation of offending agents, supportive care, reproductive assessment	Long-term endocrine recovery, breeding management, and monitoring of reproductive function
Persistent genital arousal disorder (PGAD)	Rare reproductive disorder with emergency presentation	Supportive care, fluid therapy, analgesia, management of hyperthermia and systemic complications	No established definitive treatment; symptomatic management based on clinical presentation

Emergency stabilization refers to the immediate measures required to preserve animal welfare, life, or reproductive function before definitive therapy. Definitive treatment refers to the primary medical or surgical intervention directed at correcting the underlying condition.

**Table 3:** Summary of the available evidence supporting diagnosis, treatment, and prognosis of reproductive emergencies and urgent reproductive disorders in male dromedary camels.

Condition	Camel-Specific Evidence	Extrapolated Evidence	Evidence Type	Diagnostic Certainty	Treatment Evidence	Prognostic Evidence
Testicular Trauma	Yes	Limited	Case reports and clinical studies	High	Moderate	Moderate
Testicular Torsion	Limited	Equine and bovine literature	Case reports	High	Moderate	Limited
Orchitis and Epididymitis	Yes	Limited	Clinical studies and case reports	High	Moderate	Moderate
Penile Hematoma	Limited	Equine and bovine literature	Case reports	High	Moderate	Limited
Penile and Preputial Trauma	Yes	Limited	Clinical reports and case observations	High	Moderate	Limited
Phimosis	Yes	Limited	Clinical studies and case reports	High	Moderate	Moderate
Paraphimosis	Yes	Equine literature	Clinical studies and case reports	High	Moderate	Moderate
Priapism	Limited	Equine and human literature	Case reports	Moderate–High	Limited	Limited
Urethral Obstruction	Yes	Small-ruminant and bovine literature	Clinical studies and case reports	High	Moderate	Moderate
Post-Castration Emergencies	Limited	General veterinary surgical literature	Clinical observations and case reports	High	Moderate	Limited
Acute Scrotal Hernia	Limited	Equine and bovine literature	Case reports	High	Moderate	Limited
Persistent Frenulum	Limited	Bovine, canine, and feline literature	Clinical observations and case reports	High	High	High after surgical correction
Pizzle Rot	Limited	Bovine literature	Clinical observations and microbiological findings	Moderate	Limited–Moderate	Limited
Persistent Genital Arousal Disorder (PGAD)	No published camel reports	Human medical literature	Authors’ clinical observation	Moderate	Very Limited	Unknown
Performance-Enhancing Medications and Hormonal Treatments	Limited	Human and veterinary endocrinology literature	Experimental studies and clinical observations	Moderate	Limited	Limited

## Data Availability

The raw data supporting the conclusions of this article will be made available by the authors, without undue reservation.
